# Unconventional Nickel Nitride Enriched with Nitrogen Vacancies as a High‐Efficiency Electrocatalyst for Hydrogen Evolution

**DOI:** 10.1002/advs.201800406

**Published:** 2018-06-20

**Authors:** Bin Liu, Bin He, Hui‐Qing Peng, Yufei Zhao, Junye Cheng, Jing Xia, Jianhua Shen, Tsz‐Wai Ng, Xiangmin Meng, Chun‐Sing Lee, Wenjun Zhang

**Affiliations:** ^1^ Center of Super‐Diamond and Advanced Films (COSDAF) & Department of Materials Science and Engineering City University of Hong Kong Tat Chee Avenue Kowloon Hong Kong China; ^2^ College of New Materials and New Energies Shenzhen Technology University Shenzhen 515118 Guangdong China; ^3^ Department of Chemistry Institute for Advanced Study Institute of Molecular Functional Materials and Division of Biomedical Engineering The Hong Kong University of Science & Technology Clear Water Bay Kowloon Hong Kong China; ^4^ State Key Laboratory of Chemical Resource Engineering Beijing University of Chemical Technology Beijing 100029 China; ^5^ Technical Institute of Physics and Chemistry Chinese Academy of Sciences Beijing 100190 China; ^6^ Center of Super‐Diamond and Advanced Films (COSDAF)& Department of Chemistry City University of Hong Kong Tat Chee Avenue Kowloon Hong Kong China

**Keywords:** electrocatalysis, hydrogen evolution, nitrogen vacancies, plasma‐enhanced nitridation

## Abstract

Development of high‐performance and cost‐effective non‐noble metal electrocatalysts is pivotal for the eco‐friendly production of hydrogen through electrolysis and hydrogen energy applications. Herein, the synthesis of an unconventional nickel nitride nanostructure enriched with nitrogen vacancies (Ni_3_N_1−_ *_x_*) through plasma‐enhanced nitridation of commercial Ni foam (NF) is reported. The self‐supported Ni_3_N_1−_ *_x_*/NF electrode can deliver a hydrogen evolution reaction (HER) activity competitive to commercial Pt/C catalyst in alkaline condition (i.e., an overpotential of 55 mV at 10 mA cm^−2^ and a Tafel slope of 54 mV dec^−1^), which is much superior to the stoichiometric Ni_3_N, and is the best among all nitride‐based HER electrocatalysts in alkaline media reported thus far. Based on theoretical calculations, it is further verified that the presence of nitrogen vacancies effectively enhances the adsorption of water molecules and ameliorates the adsorption–desorption behavior of intermediately adsorbed hydrogen, which leads to an advanced HER activity of Ni_3_N_1−_ *_x_*/NF.

Hydrogen, a clean and sustainable energy vector, is a promising alternative to traditional fossil fuels, and its utilization has significant value for addressing the energy crisis and environmental issues.^1^ Among the approaches developed thus far for hydrogen production, water electrolysis has demonstrated its inherent superiority in the views of its low cost and environmental benignity. In water electrolysis, electrocatalysts play a predominant role in achieving a high energy conversion efficiency in hydrogen evolution reaction (HER).^2^ However, the state‐of‐the‐art catalysts for HER are currently restricted to noble‐metal (such as Pt)–based materials, and the high cost and scarcity of these materials largely hamper their widespread applications. The development of new cost‐efficient electrocatalysts with their performance competitive to that of the precious metal‐based electrocatalysts has thus important application value.

Inspired by the great abundance (90 ppm in nature), low price (4000 times less expensive than platinum on a mole basis), and special d‐orbit electron configuration of nickel,^3^ a variety of nickel‐based compounds, including oxides, sulfides, carbides, and selenides, have been extensively studied, and their pronounced HER catalytic activities have been demonstrated.^4^ The recent theoretical and experimental results also suggested that transition metal nitrides (TMNs) could be another type of potential HER electrocatalysts due to their high binding capabilities for the adsorbates (atomic hydrogen, proton, or water molecule) and relatively low electrical resistance.^5^ Indeed, the Ni‐based TMNs in diverse nanostructures and chemical compositions, such as polycrystalline Ni_3_N film,^6^ Ni_3_N nanospheres,[[qv: 3d]] Ni_3_FeN nanoparticles,^7^ NiMoN*_x_* nanosheets,^8^ and porous hierarchical Ni_0.2_Mo_0.8_N,^9^ have been reported for HER applications. Nevertheless, their catalytic performance, e.g., hydrogen production yield, overpotential for hydrogen evolution, and stability, still needs to be improved to compete with the noble metal catalysts.

In this paper, we report for the first time that nickel nitride nanostructure synthesized directly on Ni foam substrate (Ni_3_N_1−_ *_x_*/NF) could be used as a cost‐effective self‐supported HER electrocatalyst with excellent performance. In contrast to the conventional chemical approaches, which employed hazardous nitrogen sources (such as azides, hydrazine, cyanamide, and ammonia) to synthesize metal nitrides at temperatures usually >400 °C and with long reaction duration (usually >1 h),^5^, ^7^, ^10^ the Ni_3_N_1−_ *_x_* nanostructures were formed through nitridation of commercially available Ni foam in nitrogen plasma generated by microwave. The rich energetic ions and excited neutral particles in the plasma enabled the quick synthesis of nickel nitride at reduced temperature and without the need of toxic substances.^11^ In particular, the plasma‐assisted nitridation led to the formation of significant nitrogen vacancies in nickel nitride, which was demonstrated to enhance the adsorption of water molecules (i.e., reducing kinetic energy barriers of the Volmer and Heyrovsky steps) and to ameliorate the adsorption–desorption behavior of intermediately adsorbed hydrogen on its surface. Moreover, the intimate contact between the metallic Ni_3_N_1−_ *_x_* and Ni substrate allowed fast charge transport during the HER process. As a result, the Ni_3_N_1−_ *_x_*/NF cathode presented an HER activity comparable to that of Pt/C electrode with an overpotential of 55 mV at 10 mA cm^−2^ and a Tafel slope of 54 mV dec^−1^ achieved in alkaline environment, and the cathode also showed outstanding long‐term durability toward HER.

In our experiments, a piece of clean Ni foam was subjected to the nitrogen plasma initiated by microwave for the in situ growth of nickel nitride nanostructures (**Figure** **1**a). The microwave power was 450 W, the substrate temperature was maintained at 300 °C, and the duration for plasma treatment was 90 s. The pristine Ni foam had a macroporous structure with the pore size ranging from 100 to 400 µm (Figure S1, Supporting Information), and its skeleton had a smooth surface with visible grain boundaries, as shown by the scanning electron microscopy (SEM) image in Figure 1b. After plasma treatment, the color of the Ni foam changed to dark gray (Figure S2, Supporting Information); its porous structure is still maintained, and energy‐dispersive X‐ray spectroscopy (EDX) elemental mapping verified that N was uniformly distributed on the NF surface (Figure S3, Supporting Information). The skeleton surface became rough (Figure 1c), and close observation by transmission electron microscopy (TEM) revealed that a low‐density layer with a thickness of about 700 nm was formed on Ni foam during plasma treatment (Figure 1d). The layer was identified to be Ni_3_N with enriched nitrogen vacancies by the chemical composition characterization as shown below (denoted as Ni_3_N_1−_ *_x_* hereafter), and the Ni_3_N_1−_ *_x_* was in nanoparticle configuration with a size of tens of nanometers (Figure 1e), Figure S4 (Supporting Information) and Figure 2. In the high‐resolution TEM (HRTEM) image of a nanoparticle in Figure 1f, the denoted lattice fringes with an interplanar spacing of 0.41 nm and an interfacial angle of 60° were indexed to (10$\bar{1}$0) and (01$\bar{1}$0) planes of Ni_3_N_1−_ *_x_*, and the corresponding fast Fourier transform (FFT) pattern also agreed with the diffraction pattern along the [0001] zone axis of hexagonal Ni_3_N_1−_ *_x_* (Figure 1g).

**Figure 1:**
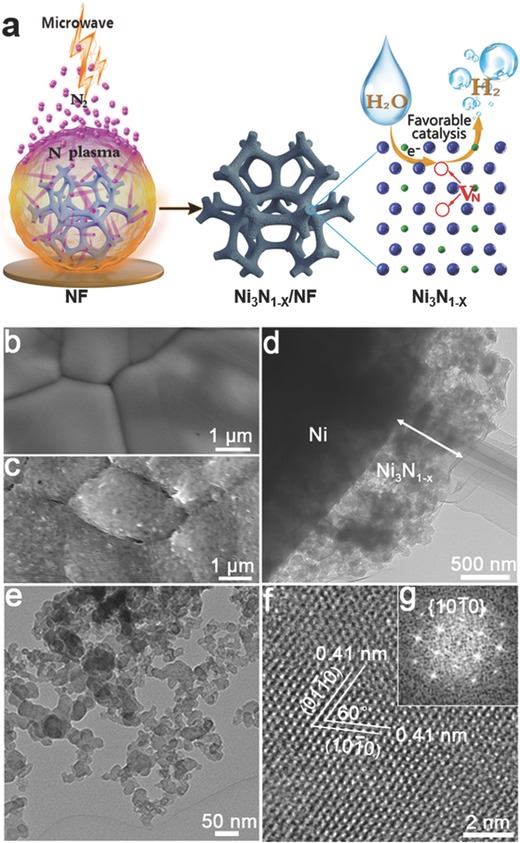
a) The scheme showing the synthesis of Ni_3_N_1−_ *_x_*/NF by subjecting Ni foam to the nitrogen plasma initiated by microwave, and the hydrogen evolution process enhanced by the presence of nitrogen vacancies. Blue: Ni atom, and green: N atom. SEM images of b) untreated Ni foam and c) Ni foam treated at N_2_ plasma. d) TEM image of Ni_3_N_1−_ *_x_* layer on Ni foam. e) TEM image of Ni_3_N_1−_ *_x_* nanoparticles scratched from Ni foam. f) HRTEM image and g) the corresponding FFT pattern of Ni_3_N_1−_ *_x_*.

To reveal the difference of the Ni_3_N_1−_ *_x_* synthesized by plasma‐enhanced nitridation, a reference nickel nitride sample was prepared by heating NF in ammonia atmosphere at 450 °C for 1 h (denoted as Ni_3_N/NF). The X‐ray diffraction (XRD) patterns (Figure S4, Supporting information) verified the formation of hexagonal Ni_3_N (JCPDS: 10‐0280) in both samples. However, the Ni_3_N_1−_ *_x_*/NF showed weaker and broader diffraction in comparison with the reference sample, indicating a lower crystallinity or a more defective structure of the sample prepared by plasma treatment. X‐ray photoelectron spectroscopy (XPS) was further performed to study the chemical composition of the two samples. In the Ni 2p XPS spectra (**Figure** **2**a), two peaks at 853.6 and 871.4 eV for Ni_3_N/NF were observed, which were assigned to the 2p_3/2_ and 2p_1/2_ of Ni^+^, respectively;^12^ and the “shake up” satellites were also seen on the higher binding energy side of the main Ni 2p peaks. In comparison, two additional peaks at 851.5 eV (2p_3/2_) and 869.5 eV (2p_1/2_), which were attributed to the existence of the less valence state of Ni (Ni^<1+^), could be resolved in the Ni 2p XPS spectra of Ni_3_N_1−_ *_x_*/NF. The predominance of Ni^<1+^ in Ni_3_N_1−_ *_x_*/NF suggested that the electron density of a considerable fraction of Ni atoms was affected by the existence of nitrogen vacancies. Moreover, the peak at 398.0 eV ascribed to the N—Ni bonding was observed for both Ni_3_N/NF and Ni_3_N_1−_ *_x_*/NF in the high‐resolution N 1s XPS spectra (Figure 2b),^13^ and a peak centered at around 399.9 eV (denoted as V_N_) was also revealed for Ni_3_N_1−_ *_x_*/NF. The observation of this extra peak at higher binding energy suggested the reduction of negative charges of nitrogen atoms and further verified the formation of nitrogen vacancies in Ni_3_N_1−_ *_x_*/NF, similar to the variation of XPS signals of oxygen in the oxide nanomaterials with oxygen vacancies.^14^ In addition, detailed compositional analysis revealed that the atomic ratio of N:Ni in Ni_3_N/NF was ≈1:3.16, which was close to the stoichiometry of Ni_3_N. By contrast, a significantly smaller atomic ratio of N:Ni (1:5.28) was obtained for Ni_3_N_1−_ *_x_*/NF, which indicated the presence of abundant nitrogen vacancies in Ni_3_N_1−_ *_x_*. All of the above characterizations revealed that the nickel nitride prepared by the microwave‐initiated nitrogen plasma treatment had a defective structure enriched with nitrogen vacancies. Moreover, it was found that the amount of nitrogen vacancies was dependent on the temperature of nitrogen plasma treatment. Herein, besides Ni_3_N_1−_ *_x_*/NF prepared at 300 °C (denoted as Ni_3_N‐300/NF for convenience), additional two nickel nitrides were prepared through nitridation of Ni foams in nitrogen plasma generated by microwave at 350 and 400 °C, and the obtained electrocatalysts were denoted as Ni_3_N‐350/NF and Ni_3_N‐400/NF, respectively. The samples were characterized using XPS. It was revealed that the atomic ratio of N:Ni gradually increased with the increment of temperature (1:5.28 for Ni_3_N‐300, 1: 4.36 for Ni_3_N‐350, and 1:3.21 for Ni_3_N‐400). Furthermore, Figure S5 (Supporting Information) shows the high‐resolution N 1s spectra and their deconvolution of the samples. The intensity of the peak centered at around 399.9 eV was associated with the amount of nitrogen vacancies. It was revealed clearly that Ni_3_N‐300/NF displayed a very distinct peak at 399.9 eV, indicating the existence of massive nitrogen vacancies. With the increase of the plasma treatment temperature, the intensity of this peak decreased and almost vanished at the temperature of 400 °C, which suggested the formation of nearly stoichiometric Ni_3_N. Based on this observation, the formation mechanism of nitrogen vacancies could be discussed as follows. In nitrogen plasma, there are molecular, atomic, and ionic nitrogen (N^+^ and N^2+^) species.^11^ Considering that the nickel nitride is an interstitial compound, in which planes of nickel atoms stack in an ABAB fashion within the unit cell, and nitrogen atoms as interstices atoms occupy the octahedral sites of the nickel lattice in an ordered fashion to minimize the repulsive N–N interactions,^15^ the nitrogen atoms of the as‐produced nitrogen species as interstices atoms permeate into the nickel lattice of Ni foam substrate surface to result in the formation of nickel nitride phase. Higher processing temperature will result in higher diffusion rate and stronger reactivity of nitrogen species, and therefore more nitrogen atoms can be filled into the nickel lattices, forming the nickel nitride phase close to perfect crystalline structure with a N/Ni ratio of 1/3 (e.g., the Ni_3_N‐400/NF). In contrast, lower processing temperature restrains the diffusion rate and reactivity of nitrogen species, leading to the formation of nickel nitride phase with nitrogen vacancies.

**Figure 2:**
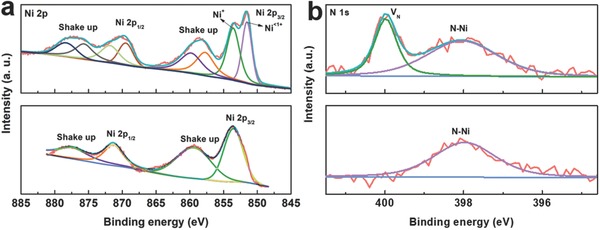
The high‐resolution XPS spectra of a) Ni 2p and b) N 1s (top: Ni_3_N_1−_ *_x_*/NF; bottom: Ni_3_N/NF).

The obtained Ni_3_N_1−_ *_x_*/NF was directly utilized as a self‐supported cathode for hydrogen generation in a 1.0 m KOH solution (pH 14) using a standard three‐electrode configuration. To highlight the superiority of Ni_3_N_1−_ *_x_*/NF, the catalytic performance of bare NF, Ni_3_N/NF, and commercial Pt/C (20 wt% Pt/XC‐72) was also evaluated for comparison. **Figure** **3**a presents the linear sweep voltammetry (LSV) curves of all these samples. Among them, Pt/C/NF showed the best HER activity with an overpotential (η_10_) of 46 mV at 10 mA cm^−2^, which was consistent with those of the previous reports.^16^ Impressively, Ni_3_N_1−_ *_x_*/NF electrode exhibited an electrocatalytic performance very competitive to that of Pt/C/NF electrode, i.e., an onset potential close to that of commercial Pt/C and an η_10_ of 55 mV (only 9 mV higher than that of Pt/C/NF). The η_10_ value of Ni_3_N_1−_ *_x_*/NF was substantially reduced as compared with that of Ni_3_N/NF (140 mV), implying the decisive role of nitrogen vacancies in enhancing the HER activity of nickel nitrides. Figure 3b presents the Tafel slopes of the samples derived from the polarization curves at a slow scan rate of 1 mV s^−1^. Ni_3_N_1−_ *_x_*/NF showed a Tafel slope of 54 mV dec^−1^, which was slightly higher than that of Pt/C/NF (45 mV dec^−1^) but obviously smaller than that of Ni_3_N/NF (96 mV dec^−1^). Because the Tafel slope is directly associated with the HER reaction kinetics of electrocatalyst,^17^ the lower Tafel slope of Ni_3_N_1−_ *_x_*/NF indicates that the presence of nitrogen vacancies results in its faster kinetics and superior catalytic activity as compared with Ni_3_N/NF. In addition, Figure S6 (Supporting Information) shows the LSV curves and the corresponding Tafel plots of the samples prepared at different temperatures of plasma treatment. It was revealed that Ni_3_N‐300/NF (i.e., Ni_3_N_1−_ *_x_*/NF) with the largest amount of nitrogen vacancies exhibited the smallest overpotential and Tafel slope among the samples. These results verified that the HER catalytic activity of the as‐prepared samples was closely related to the amount of nitrogen vacancies, highlighting the importance of nitrogen vacancies. As summarized in Figure 3c and Table S1 (Supporting Information), Ni_3_N_1−_ *_x_*/NF has actually the lowest η_10_ in all nitride‐based HER electrocatalysts in alkaline media reported thus far, e.g., Ni_3_N nanospheres (≈185 mV),[[qv: 3d]] Ni_3_FeN nanosheets/NF (75 mV),^18^ Co–Ni_3_N nanorods (194 mV),^19^ hierarchical NiMoN/carbon cloth (109 mV),^9^ CoN nanowires (97 mV),^20^ and Co_5.47_N@N–C (149 mV)^21^; and the overall performance of Ni_3_N_1−_ *_x_*/NF is also among the best non‐noble‐metal HER catalysts working in basic electrolytes including hydroxides, sulfides, carbides, phosphides, and selenides (Table S2, Supporting Information). Another critical factor to evaluate an HER catalyst is its long‐term stability. To explore the durability of Ni_3_N_1−_ *_x_*/NF as a self‐supported cathode, a fixed overpotential of 100 mV was applied to Ni_3_N_1−_ *_x_*/NF. As shown in the inset of Figure 3d, the current density maintained almost unchanged during the 50 h tests. Moreover, the polarization curve recorded after the durability test almost overlapped with the initial one before the test, and the overpotential required to achieve a current density of 100 mA cm^−2^ merely increased by 6 mV, demonstrating its excellent catalytic stability in basic condition.

**Figure 3:**
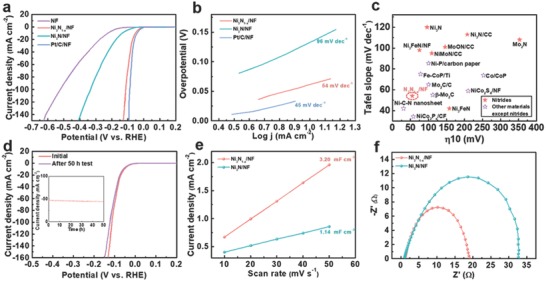
a) The LSV curves of NF, Ni_3_N_1−_ *_x_*/NF, Ni_3_N/NF, and Pt/C/NF measured in 1.0 m KOH solution (pH 14). b) Corresponding Tafel plots for the samples. c) The comparison of the performance of Ni_3_N_1−_ *_x_*/NF with the previously reported nitrides and other non‐noble‐metal‐based electrocatalysts in basic environment (the related references are listed in Tables S1 and S2 in the Supporting Information). d) LSV curves before and after the stability test for 50 h. The inset is the chronoamperometry curve of Ni_3_N_1−_ *_x_*/NF recorded at an overpotential of 100 mV for a total duration of 50 h. e) The linear fitting of the capacitive currents of the electrodes as a function of scan rates for Ni_3_N_1−_ *_x_*/NF and Ni_3_N/NF. f) Nyquist plots of Ni_3_N_1−_ *_x_*/NF and Ni_3_N/NF at an overpotential of 120 mV from 100 kHz to 10 mHz.

To understand the effects of nitrogen vacancies on the superior HER activity of Ni_3_N_1−_ *_x_*/NF to that of Ni_3_N/NF, we evaluated their electrochemically active surface areas (EASAs) by measuring electrochemical double‐layer capacitance (*C*_dll_).[[qv: 4a,22]] As demonstrated in Figure 3e, the *C*_dl_ value of Ni_3_N_1−_ *_x_*/NF (3.20 mF cm^−2^) was almost threefold higher than that of the Ni_3_N/NF (1.14 mF cm^−2^), which indicated that Ni_3_N_1−_ *_x_*/NF had much increased electrochemically active sites. In addition, electrochemical impedance spectroscopy (EIS) was also carried out to study the HER kinetics of Ni_3_N_1−_ *_x_*/NF and Ni_3_N/NF, as shown in Figure 3f. It was obvious that Ni_3_N_1−_ *_x_*/NF had a much smaller charge transfer resistance (*R*_ct_) at the interface between the electrode and electrolyte than that of Ni_3_N/NF (18.1 vs 31.8 Ω), illustrating a highly efficient and fast electron transport in the HER process in Ni_3_N_1−_ *_x_*/NF.^23^ The results agreed well with the observation of smaller Tafel slope and superior HER kinetics of Ni_3_N_1−_ *_x_*/NF. Electrocatalytic HER is a representative surface reaction, and the surface wettability of an electrocatalyst is directly associated with its capability for the access of electrolyte, the adsorption of water molecules, and the electrocatalytic activity. In this work, we also measured the water contact angles on Ni_3_N_1−_ *_x_*/NF and Ni_3_N/NF. The smaller contact angle on Ni_3_N_1−_ *_x_*/NF (91.3° vs 128.2° for Ni_3_N/NF), as illustrated in Figure S7 in the Supporting Information, suggested its better wettability, which would benefit the adsorption of water and the enhancement of HER reaction kinetics of Ni_3_N_1−_ *_x_*/NF.^24^

By normalizing the HER current densities with respect to the EASAs, the intrinsic activities of Ni_3_N_1−_ *_x_*/NF and Ni_3_N/NF were obtained, as depicted in Figure S8 (Supporting Information). At a given potential after onset, the current density of Ni_3_N_1−_ *_x_*/NF was considerably higher than that of the Ni_3_N/NF, implying that Ni_3_N_1−_ *_x_*/NF had a significantly improved intrinsic HER activity. Density functional theory (DFT) simulations were carried out to pinpoint the origin of the enhanced intrinsic activity of Ni_3_N_1−_ *_x_*/NF. As shown in **Figure** **4**a, with the existence of nitrogen vacancies, a continuous distribution of the density of states (DOS) and a large number of electronic states near the Fermi level were observed, suggesting that Ni_3_N_1−_ *_x_* was still in the metallic state with a high electrical conductivity. The results were consistent with the EIS measurements that Ni_3_N_1−_ *_x_*/NF had fast electron transport in the electrocatalytic process. Furthermore, as revealed by the calculated partial charge density distribution in Figure 4b and Figure S9 (Supporting Information), the existence of nitrogen vacancy might lead to charge redistribution in Ni_3_N_1−_ *_x_*, in which the electron density around Ni atoms next to the nitrogen vacancy substantially increased. Such a charge redistribution led to the formation of Ni^<1+^, as revealed in the XPS measurements. Moreover, the change of electronic state of Ni_3_N_1−_ *_x_* relative to Ni_3_N could be reflected by ultraviolet photoelectron spectroscopy (UPS), as shown in Figure S10 (Supporting Information). The valence band maximum (VBM) value of Ni_3_N was determined to be ≈3.4 eV. For Ni_3_N_1−_ *_x_* with nitrogen vacancies, the VBM increased to 4.1 eV, which indicated the VBM of Ni_3_N_1−_ *_x_* shifted more from the Fermi level than that of Ni_3_N due to the presence of nitrogen vacancies. The shift of the valence band implied the d‐band center of Ni_3_N_1−_ *_x_* also shifted as compared to that of Ni_3_N, and the downshift of the d‐band center of Ni_3_N_1−_ *_x_* would facilitate the desorption of H from its surface, thus enabling an enhanced HER activity of Ni_3_N_1−_ *_x_*/NF.^25^

**Figure 4:**
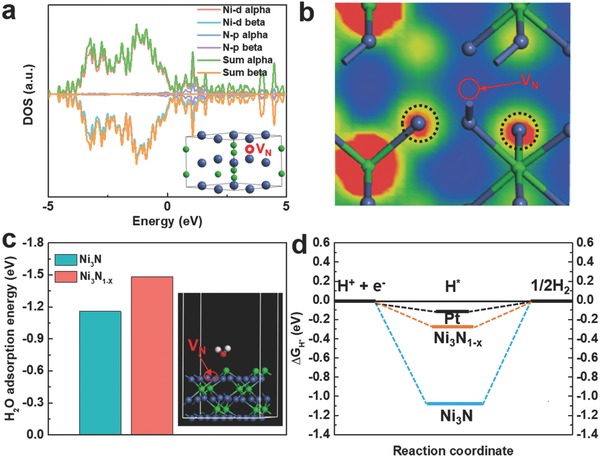
a) Total and partial electronic density of states (TDOS and PDOS) calculated for Ni_3_N_1−_ *_x_*. The Fermi level is set at 0 eV. The inset shows the atomic structure model of Ni_3_N_1−_ *_x_*. b) Partial charge density distribution of Ni_3_N_1−_ *_x_*. c) Adsorption energies of H_2_O molecules on the surfaces of Ni_3_N and Ni_3_N_1−_ *_x_*. The inset is a side‐view schematic model showing the Ni_3_N_1−_ *_x_* structure with a H_2_O molecule adsorbed on its surface. d) The calculated free‐energy diagram of HER at the equilibrium potential for Ni_3_N, Ni_3_N_1−_ *_x_*, and Pt reference. H* denotes that intermediate adsorbed hydrogen.

For the HER in basic media, two separate pathways (the Volmer–Tafel and the Volmer–Heyrovsky mechanism) have been proposed for reducing H* to H_2_. Specifically, these two distinct mechanisms involve three principal steps, referring to the Volmer (adsorption and electrochemical reduction of water: H_2_O + e → H* + OH^−^), the Heyrovsky (electrochemical desorption: H* + H_2_O + e → H_2_ + OH^−^), and the Tafel (chemical desorption: H* + H* → H_2_) reactions.^26^ Tafel plot analysis is generally utilized to elucidate the reaction mechanism, which may provide information associated with the rate‐determining steps.^27^ The Tafel slopes of 54 and 96 mV dec^−1^ for Ni_3_N_1−_ *_x_*/NF and Ni_3_N/NF implied a Volmer–Heyrovsky mechanism on the two electrocatalysts,^28^ where the adsorption of H_2_O molecules is fundamental in both reactions. Therefore, we calculated the adsorption energies of H_2_O molecules on the surfaces of Ni_3_N_1−_ *_x_* and Ni_3_N.^29^ The optimized structures of Ni_3_N_1−_ *_x_* and Ni_3_N with H_2_O molecules adsorbed on their surfaces are shown in Figure 4c and Figure S11 (Supporting Information), respectively. The Ni_3_N_1−_ *_x_* enriched with nitrogen vacancies possessed an increased adsorption energy (absolute value) as compared with the stoichiometric Ni_3_N (1.48 vs 1.15 eV as summarized in Figure 4c), verifying that the presence of nitrogen vacancies could decrease the energy barrier for the adsorption of H_2_O. As a result, the Volmer step and Heyrovsky step could be promoted simultaneously, which greatly accelerated HER kinetics of Ni_3_N_1−_ *_x_*/NF. On the other hand, HER activity is also strongly related to the Gibbs free energy $\left(|\Delta G_{H^{*}}|\right)$ of the intermediate adsorbed hydrogen, and $\left|\Delta G_{H^{*}}\right|$ value is regarded as a descriptor of HER activity for a catalyst, i.e., a smaller $\left|\Delta G_{H^{*}}\right|$ enables better activity toward HER, and an optimal HER activity can be achieved at $\left|\Delta G_{H^{*}}\right|$ = 0.0 eV due to the balanced proton reduction rate and the removal of adsorbed hydrogen from the catalyst surface.^30^ In this work, we also used DFT to calculate the $\left|\Delta G_{H^{*}}\right|$ value on the surface of Ni_3_N with and without nitrogen vacancies, as shown in Figure 4d. It was revealed that Ni_3_N_1−_ *_x_* had a substantially reduced $\left|\Delta G_{H^{*}}\right|$ value (0.28 eV) compared to the Ni_3_N (1.05 eV), which illustrated that the presence of nitrogen vacancies induced a more favorable adsorption–desorption behavior of intermediately adsorbed hydrogen H* on Ni_3_N_1−_ *_x_*. The theoretical simulations also agreed well with the experimental observations that Ni_3_N_1−_ *_x_*/NF had an obviously improved HER catalytic activity (including significantly reduced overpotential and Tafel slope) comparable to the Ni_3_N/NF in basic condition. In addition, the favorable adsorption–desorption behavior of H* initiated by nitrogen vacancies also led to obviously enhanced HER activity of Ni_3_N_1−_ *_x_*/NF in neutral electrolyte. As shown in Figures S12 and S13 (Supporting Information), Ni_3_N_1−_ *_x_*/NF displayed an η_10_ value of only 89 mV and a Tafel slope of 63 mV dec^−1^, respectively, with outstanding durability, both of which were much smaller than those of Ni_3_N/NF without nitrogen vacancies (223 mV and 106 mV dec^−1^, respectively).

Based on the structural analysis and the theoretical simulation, the outstanding catalytic performance of the Ni_3_N_1−_ *_x_*/NF electrode could be mainly attributed to collective effects of the following aspects: 1) the nitrogen vacancies optimized the electronic structure of Ni_3_N_1−_ *_x_*, which, on one hand, reduced the energy barrier for the adsorption of H_2_O (promoting the Volmer step and Heyrovsky step simultaneously), and, on the other hand, induced balanced adsorption–desorption of intermediate adsorbed hydrogen H* on Ni_3_N_1−_ *_x_*. 2) The intrinsic metallicity of the Ni_3_N_1−_ *_x_* layer synthesized by plasma nitridation guaranteed the fast charge transfer on the interface between active material and electrolyte during catalytic process. 3) The direct growing of Ni_3_N_1−_ *_x_* on NF formed a robust integrated electrode without the use of polymer binders, which avoided the shelter of active sites and provided a pathway for fast electron transportation.^31^ Moreover, the strong adhesion of Ni_3_N_1−_ *_x_* layer on Ni foam also benefited its mechanical and catalytic stabilities.

In conclusion, a self‐supported cathode comprising Ni_3_N_1−_ *_x_* nanoparticles enriched with nitrogen vacancies was prepared by a facile nitrogen plasma treatment of NF. Ni_3_N_1−_ *_x_*/NF cathode exhibited a high catalytic activity and outstanding stability for HER in alkaline condition. Theoretical calculations verified that Ni_3_N_1−_ *_x_* was in the metallic state with a high electrical conductivity, and the presence of nitrogen vacancies facilitated the adsorption of H_2_O on Ni_3_N_1−_ *_x_* and optimized adsorption–desorption behavior of intermediately adsorbed hydrogen, which were responsible for the advanced HER catalytic activity of Ni_3_N_1−_ *_x_*/NF. The development of new integrated Ni_3_N_1−_ *_x_*/NF cathodes with the overall performance comparable to that of commercial Pt/C electrodes demonstrates a promising route to achieve highly efficient electrocatalysts for practical hydrogen evolution.

## Experimental Section

Experimental Section is available in the Supporting Information.

## Conflict of Interest

The authors declare no conflict of interest.

## Supporting information

Supplementary
