# Supplementary material for: Unconventional Nickel Nitride Enriched with Nitrogen Vacancies as a High‐Efficiency Electrocatalyst for Hydrogen Evolution
Source: Adv Sci (Weinh). 2018 Jun 20;5(8):1800406. doi: 10.1002/advs.201800406 (PMC6097009; doi:10.1002/advs.201800406)
Supplement: Supplementary file 1 — Supplementary [file ADVS-5-1800406-s001.pdf]

Copyright WILEY-VCH Verlag GmbH & Co. KGaA, 69469 Weinheim, Germany, 2018.

Supporting Information

for *Adv. Sci.,* DOI: 10.1002/advs.201800406

Unconventional Nickel Nitride Enriched with Nitrogen Vacancies as a High-Efficiency Electrocatalyst for Hydrogen Evolution

*Bin Liu, Bin He, Hui-Qing Peng, Yufei Zhao, Junye Cheng, Jing Xia, Jianhua Shen, Tsz-Wai Ng, Xiangmin Meng, Chun-Sing Lee, and Wenjun Zhang**

Supporting Information

Unconventional Nickel Nitride Enriched with Nitrogen Vacancies as A High-Efficiency Electrocatalyst for Hydrogen Evolution

*Bin Liu, Bin He, Hui-Qing Peng, Yufei Zhao, Junye Cheng, Jing Xia, Jianhua Shen, Tsz-Wai Ng, Xiangmin Meng, Chun-Sing Lee, Wenjun Zhang**

Dr. B. Liu,[†] Dr. B. He,[†] J. Cheng, Dr. J. Shen, Prof. W. Zhang

Center of Super-Diamond and Advanced Films (COSDAF) & Department of Materials Science and Engineering, City University of Hong Kong, Tat Chee Avenue, Kowloon, Hong Kong, China

E-mail: apwjzh@cityu.edu.hk

Dr. H.-Q. Peng[†]

Department of Chemistry, Institute for Advanced Study, Institute of Molecular Functional Materials and Division of Biomedical Engineering, The Hong Kong University of Science & Technology, Clear Water Bay, Kowloon, Hong Kong, China

Dr. Y. Zhao

State Key Laboratory of Chemical Resource Engineering, Beijing University of Chemical Technology, Beijing 100029, China

Dr. J. Xia, Prof. X. Meng

Technical Institute of Physics and Chemistry, Chinese Academy of Sciences, Beijing 100190, China

Dr. T.-W. Ng, Prof. C.-S. Lee

Center of Super-Diamond and Advanced Films (COSDAF) & Department of Chemistry, City University of Hong Kong, Tat Chee Avenue, Kowloon, Hong Kong, China

Dr. B. He[†]

College of New Materials and New Energies, Shenzhen Technology University, Shenzhen 515118, Guangdong, China

[†] These authors contributed equally to this work.

EXPERIMENTAL SECTION

Material synthesis. The synthesis of Ni_3N_{1-x}/NF self-supported electrocatalysts was carried out in a 1.5 kW ASTeX MPCVD system. Before the synthesis, Ni foams (Changsha Lyrun New Material Co., Ltd, China) with an area of 1.5 cm × 3.0 cm were first washed in acetone, alcohol, and deionized (DI) water. After dried with nitrogen gas, the Ni foams were placed into the MPCVD system and subjected to the nitrogen plasma for the *in-situ* growth of nickel nitride. Nitrogen plasma was produced using a microwave power of 450 W. During plasma nitridation, the pressure was maintained at 14 Torr with a nitrogen flow rate of 30 sccm, the substrate temperature was 300 ^{o}C, and the duration of treatment was 90 s. After the plasma was switched off, the samples were cooled down to room temperature naturally. For the synthesis of the control Ni_3N/NF sample, the NF was placed in a tubular furnace and heated at 450 ^{o}C for 1 h under ammonia flow.

Material characterization. X-ray diffraction (XRD; Philips X' Pert, Cu-K_α radiation) was utilized to characterize the crystal structure of the as-grown samples. X-ray photoelectron spectroscopy (XPS) and ultraviolet photoelectron spectroscopy (UPS) were performed using an ESCALAB 220i-XL spectrophotometer with Al-K_α radiation. Scanning electron microscopy (SEM) images and energy dispersive X-ray (EDX) mapping pictures of the samples were collected on a Philips XL30 FEG with the accelerating voltage of 20 kV. Transmission electron microscope (TEM) and high-resolution TEM (HRTEM) images were acquired on JEM-2100F operated at 200 kV. Contact angle measurements were conducted with a DKSH system.

Electrochemical measurements. All the electrochemical tests were carried out in a typical three-electrode system at an electrochemical station (Germany, Zahner). Linear sweep voltammetry (LSV) with a scan rate of 5 mV s^{-1} was conducted in 1 M KOH using a Ag/AgCl electrode (3 M KCl) as the reference electrode, a graphite rod plate as the counter electrode, and the sample electrocatalysts as the working electrode. Before measurements, high-purity N_2 gas was used to purge the system for at least 30 min, so as to ensure the saturation of N_2 in the electrolyte solution; and during the tests, the system was continuously purged with N_2. All the potentials in this work were calibrated to a reversible hydrogen electrode (RHE) according

to the formula E(RHE) = E(Ag/AgCl) + 0.194 + 0.05916 × pH. The electrochemical stability of the catalyst was evaluated by chronoamperometry test under a constant overpotential of 100 mV. Electrochemical impedance spectroscopy was performed when the working electrode was biased at a constant overpotential of 120 mV and the frequency was swept from 100 kHZ to 10 mHZ with a 10 mV AC dither. The impedance data were fit to a simplified circuit to extract the series and charge transfer resistances.

DFT calculations. Plane-wave density functional theory (DFT) calculations of the electronic properties of the electrocatalysts were performed using the DMOL module in Material Studio. GGA with PBE functional was used for the DFT exchange correlation energy, and 300 eV of kinetic energy cutoff was assigned to the plane-wave basis set. The self-consistent field (SCF) tolerance was 2×10^{-6} eV. The Brillouin zone was sampled by 2×2×1 k-points. The core electrons were replaced with ultrasoft pseudo-potentials. For calculations of adsorption energy, the (0001) facet was modeled with vacuum widths of 15 Å. We adopted slabs with 6 layers Ni_3N layers consisting of 48 atoms ($Ni_{36}N_{12}$). The periodically repeated slabs were separated from their neighboring images by a 15-Å-wide vacuum in the direction perpendicular to the surface.

The Gibbs free-energy (ΔG_{H*}) is expressed as:[1]

$$\Delta G_{H*} = \Delta E_{H*} + \Delta E_{ZPE} - T\Delta S$$

where ΔE_{H*} is the adsorption energy of atomic hydrogen on the given unit cell, ΔE_{ZPE} is the difference corresponding to the zero point energy between the adsorbed hydrogen and hydrogen in the gas phase, and ΔS is entropy change of H^* adsorption. As the entropy of hydrogen in absorbed state is negligible, ΔS can be calculated as -1/2 S_0 (S_0 is the entropy of H_2 in the gas phase at standard conditions, 1 bar of H_2 and pH = 0 at 300 K). Therefore the free energy of the adsorbed state can be taken as:

$$\Delta G_{H*} = \Delta E_{H*} + 0.24 \text{ eV}$$

HER active sites for electrocatalysts were performed by adding a hydrogen atom at a distance of 1.5 Å on the surface of electrocatalysts. Pt was selected as standard electrode for free energy calculation.

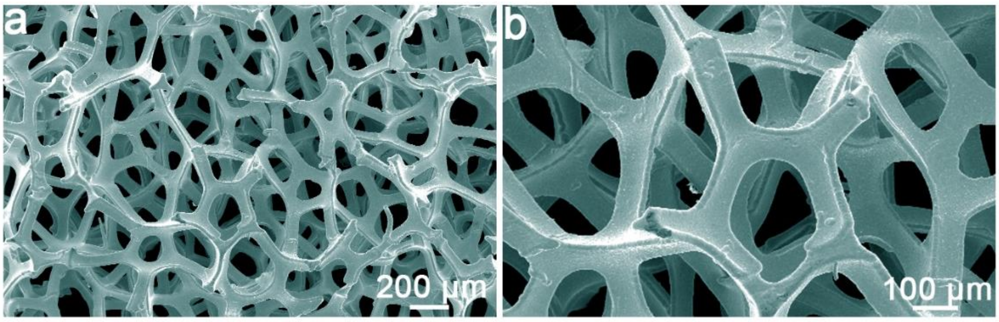

Figure S1. SEM image of a pristine Ni foam.

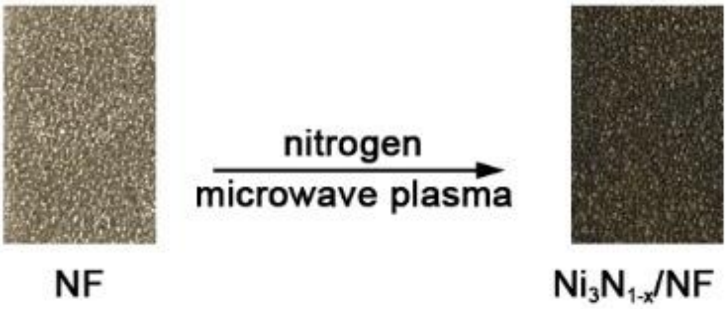

Figure S2. Photos of the NF before and after nitrogen plasma treatment.

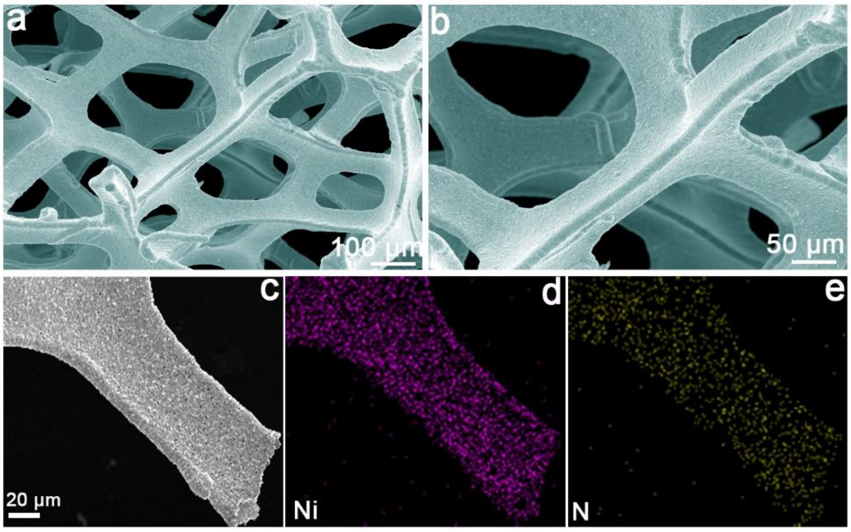

Figure S3. (a, b) Low-magnification SEM images, and (c, d, e) EDX mapping images of the NF after nitrogen plasma treatment at 300 ^{o}C for 90 s.

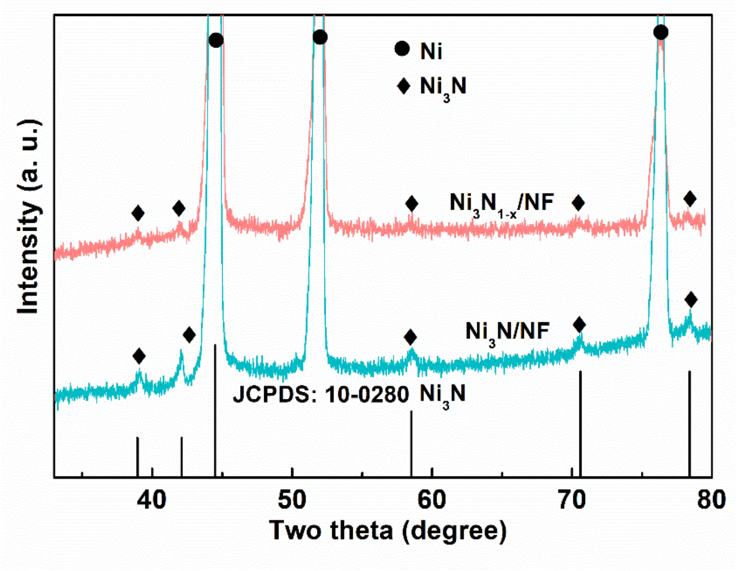

Figure S4. The XRD patterns of Ni_3N/NF and Ni_3N_{1-x}/NF.

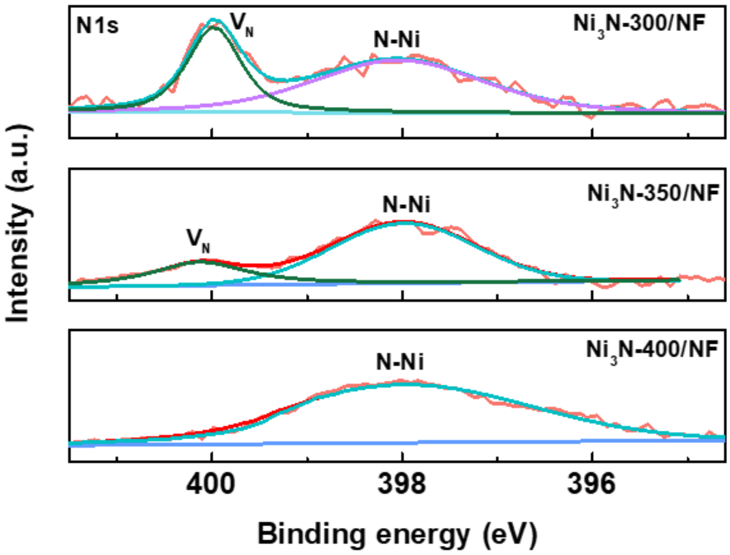

Figure S5. The high-resolution N 1s spectra and their deconvolution of Ni_3N-300/NF, Ni_3N-350/NF and Ni_3N-400/NF. Note: Ni_3N-300/NF was also denoted as the Ni_3N_{1-x}/NF in the main text of the paper.

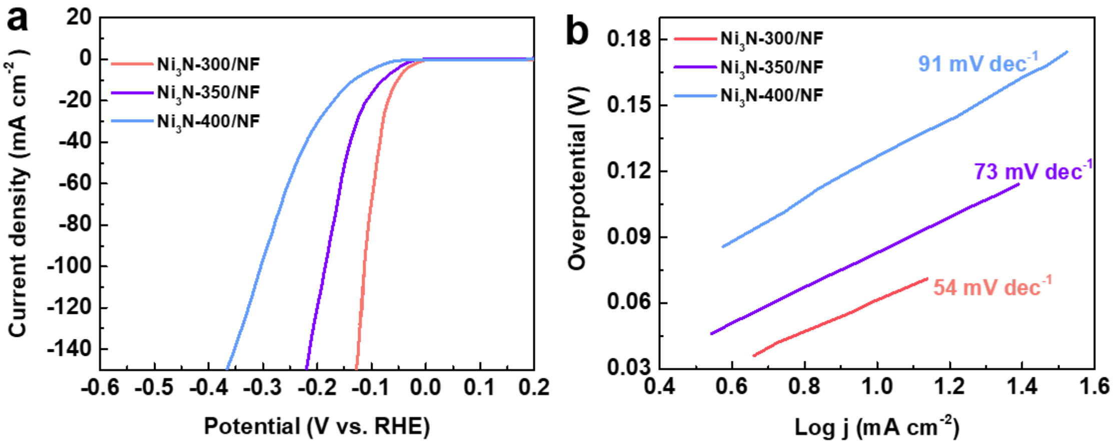

Figure S6. (a) The LSV curves of Ni_3N-300/NF, Ni_3N-350/NF and Ni_3N-400/NF measured in 1.0 M KOH solution (pH 14). (b) Corresponding Tafel plots for the samples.

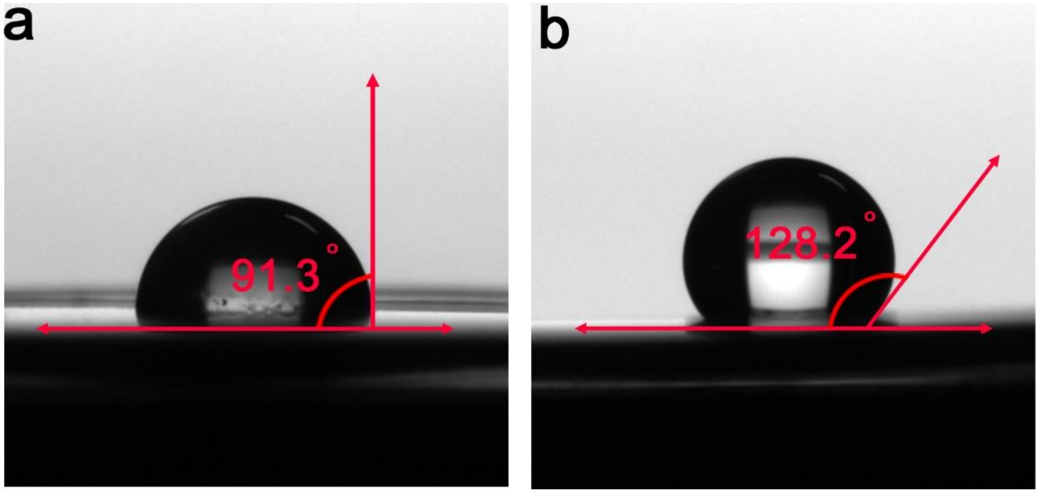

Figure S7. Water contact angle measurements for (a) Ni_3N_{1-x}/Ni foil and (b) Ni_3N/Ni foil after resting the water droplet on the surface for 4 s.

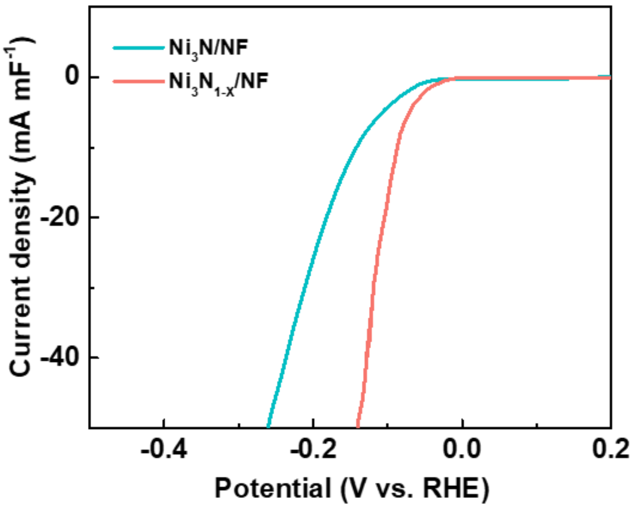

Figure S8. Polarization curves normalized by the electrochemical double-layer capacitance for Ni_3N/NF and Ni_3N_{1-x}/NF.

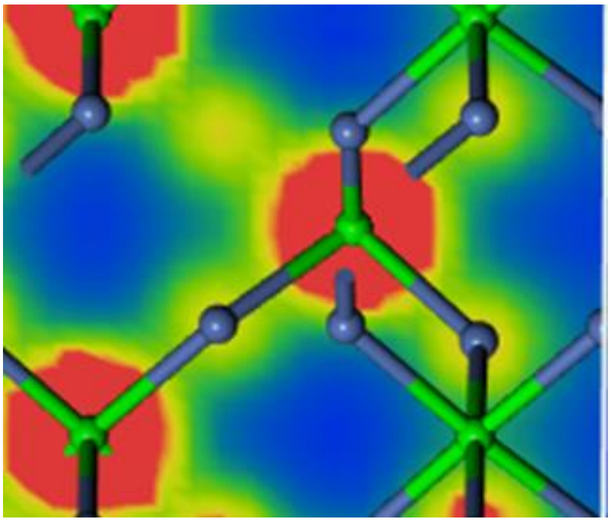

Figure S9. The calculated partial charge density of Ni_3N.

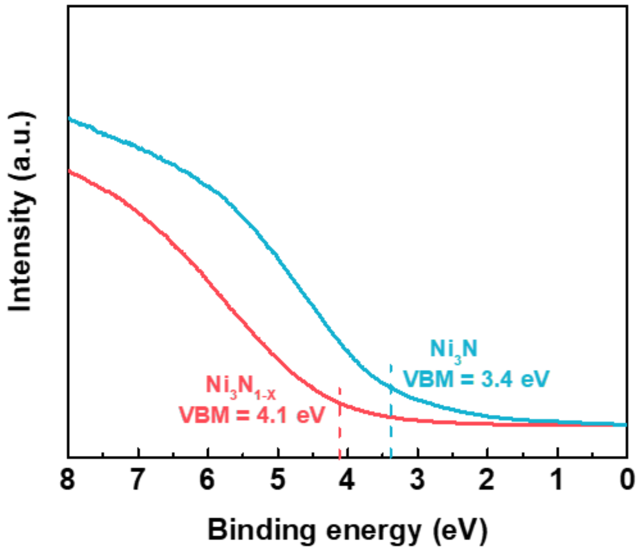

Figure S10. The UPS spectra of valence bands of Ni_3N_{1-x} and Ni_3N.

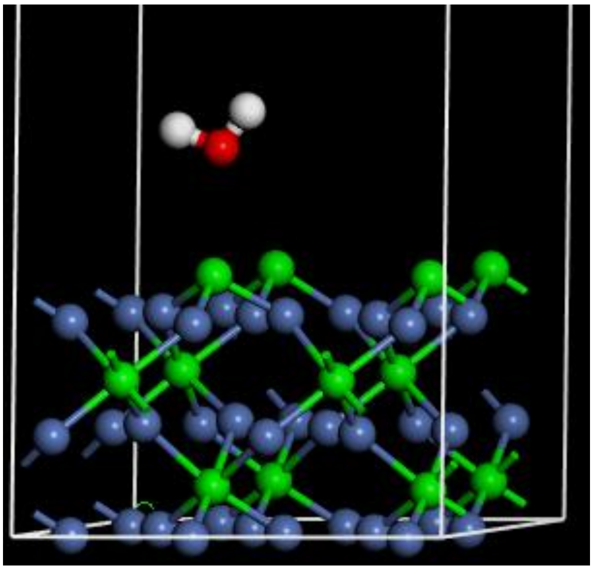

Figure S11. The side-view schematic model showing Ni_3N with H_2O molecule adsorbed on its surface.

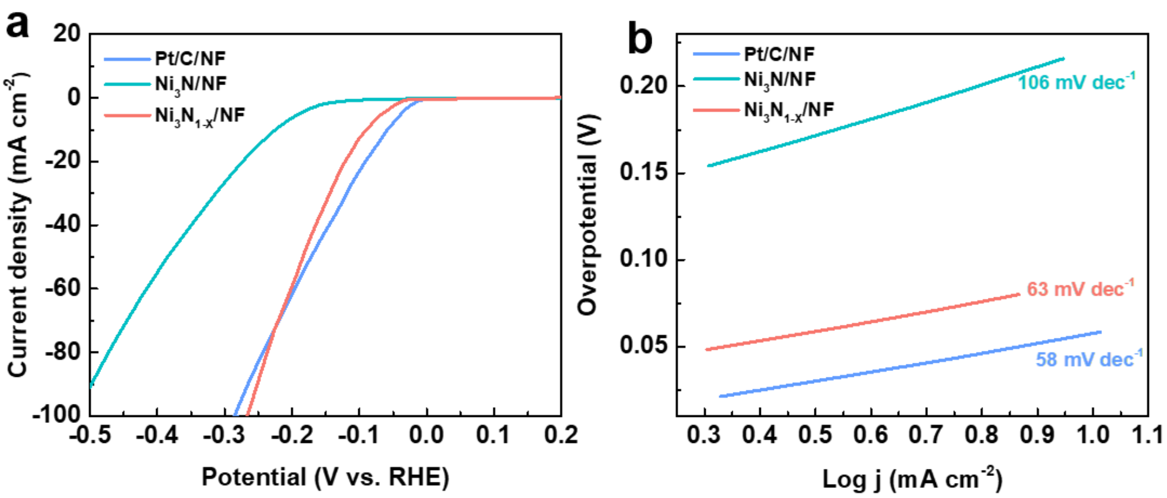

Figure S12. LSV curves and corresponding Tafel plots of Ni_3N/NF, Ni_3N_{1-x}/NF and Pt/C/NF in1.0 M PBS solution (pH 7).

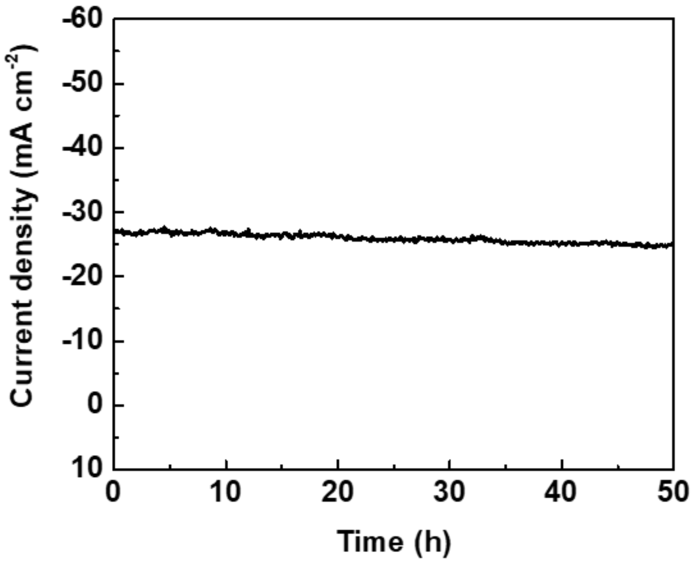

Figure S13. The chronoamperometry curve of Ni_3N_{1-x}/NF recorded at an overpotential of 150 mV for a total duration of 50 hours in 1.0 M PBS solution (pH 7).

Table S1. Comparison of the HER activity of Ni_3N_{1-x}/NF with other nitrides-based electrocatalysts in basic condition.

**Catalysts**	**Electrolyte**	**Overpotential at 10 mA cm^{-2} (mV)**	**Tafel slope (mV dec^{-1})**	**Refs.**
Ni_3N_{1-x}/NF	1.0 M KOH	55	54	This work
Ni_3FeN/NF	1.0 M KOH	75	98	*Chem. Mater.* **2016**, *28*, 6934.
Ni_3FeN	1.0 M KOH	158	42	*Adv. Energy. Mater.* **2016**, *6*, 1502585.
NiMoN/CC	1.0 M KOH	109	95	*Adv. Energy Mater.* **2016**, *6*, 1600221.
MoON/CC	1.0 M KOH	146	101	*Adv. Energy Mater.* **2016**, *6*, 1600221.
Ni_3N/CC	1.0 M KOH	208	113	*Adv. Energy Mater.* **2016**, *6*, 1600221.
Mo_2N	1.0 M KOH	353	108	*J. Mater. Chem. A* **2015**, *3*, 8361.
Ni_3N	1.0 M KOH	96	120	*J. Mater. Chem. A* **2015**, *3*, 8171.
Ni_3N/NF	1.0 M KOH	121	109	*Electrochim. Acta* **2016**, *191*, 841.
NiCoN nanowires	1.0 M KOH	145	105.2	*Energy Technol.* **2017**,*5*,1908.
CoN nanowires	1.0 M KOH	97	93.9	*Electrochim. Acta* **2018**, 273, 229.
$Co_{5.47}N$ @N-C	1.0 M KOH	149	86	*ACS Appl. Mater. Interfaces* **2018**, *10*, 7134.
Ni_3N nanorods	1.0 M KOH	305	197.5	*Adv. Mater.* **2018**, *30*, 1705516.
Co-Ni_3N nanorods	1.0 M KOH	194	156	*Adv. Mater.* **2018**, *30*, 1705516.
Ni_3N nanosphere	1.0 M KOH	~185	-	*Adv. Energy Mater.* **2017**, *7*, 1601735.

Table S2. Comparison of the HER activity of Ni_3N_{1-x}/NF with other non-noble metal-based electrocatalysts in basic condition.

Catalysts	Electrolyte	Overpotential at 10 mA cm^{-2} (mV)	Tafel slope (mV dec^{-1})	Refs.
Ni_3N_{1-x}/NF	1.0 M KOH	55	54	This work
Ni-P/carbon paper	1.0 M KOH	100	85.4	*Adv. Funct. Mater.* **2016**, *26*, 4067.
β-Mo_2C	0.1 M KOH	112	55	*Angew. Chem. Int. Ed.* **2015**, *54*, 15395.
MoC_x nano-octahedrons	1.0 M KOH	151	59	*Nat. Commum.* **2015,** *6*, 6512.
Co/CoP	1.0 M KOH	253	73.8	*Adv. Energy Mater.* **2017**, *7*, 1602355.
Ni_3S_2	1.0 M KOH	~ 220	-	*J. Am. Chem. Soc.* **2015,** *137*, 14023.
$Ni_{0.89}Co_{0.11}Se_2$ MNSN/NF	1.0 M KOH	85	52	*Adv Mater* ***2017***, *29*, 1606521
$NiCo_2S_4$ NW/NF	1.0 M KOH	210	58.9	*Adv. Funct. Mater.* **2016**, *26*, 4661.
Ni-C-N nanosheets	1.0 M KOH	30.8	~ 42	*J. Am. Chem. Soc.* **2016**, *138*, 14546.
Ni-NiO/N-rGO	1.0 M KOH	135	46	*Adv. Funct. Mater.* **2015**, *25*, 5799.
Mo_2C/carbon microflowers	1.0 M KOH	100	65	*ACS Nano* **2016**, *10*, 11337.
$NiCo_2P_x$/CF	1.0 M KOH	58	34.3	*Adv. Mater.* **2017**, *29*, 1605502.
Fe-CoP/Ti	1.0 M KOH	78	75	*Adv. Mater.* **2017**, *29*, 1602441.
Ni_2P/Fe_2P	1.0 M KOH	121	67	*Adv. Energy Mater.* **2018**, *8*,1800484.
$Co/Co_2Mo_3O_8$	1.0 M KOH	25	33	*ACS Catal.* **2018**, *8*, 5062
$Mo_{0.6}Ni_{0.4}$	1.0 M KOH	65	72	*Adv. Mater. Interfaces* **2018**, *5*, 1800359.

Co/N, B-C	1.0 M KOH	117	146	*Adv. Funct. Mater.* **2018**, *28*, 1801136.

Reference

1. a) Y. Zheng, Y. Jiao, Y. Zhu, L. H. Li, Y. Han, Y. Chen, A. Du, M. Jaroniec, S. Z. Qiao, *Nat. Commun.* **2014**, *5*, 3783; b) J. K. Nørskov, T. Bligaard, A. Logadottir, J. Kitchin, J. G. Chen, S. Pandelov, U. Stimming, *J. Electrochem. Soc.* **2005**, *152*, 23.
